# The Role of Glycation on the Aggregation Properties of IAPP

**DOI:** 10.3389/fmolb.2020.00104

**Published:** 2020-06-03

**Authors:** Giulia Milordini, Elsa Zacco, Matthew Percival, Rita Puglisi, Fabrizio Dal Piaz, Pierandrea Temussi, Annalisa Pastore

**Affiliations:** ^1^UK Dementia Research Institute at the Maurice Wohl Institute of King’s College London, London, United Kingdom; ^2^Dipartimento di Medicina, Chirurgia e Odontoiatria “Scuola Medica Salernitana”, University of Salerno, Salerno, Italy

**Keywords:** amylin, biophysics, diabetes, glycation, protein aggregation

## Abstract

Epidemiological evidence shows an increased risk for developing Alzheimer’s disease in people affected by diabetes, a pathology associated with increased hyperglycemia. A potential factor that could explain this link could be the role that sugars may play in both diseases under the form of glycation. Contrary to glycosylation, glycation is an enzyme-free reaction that leads to formation of toxic advanced glycation end-products (AGEs). In diabetes, the islet amyloid polypeptide (IAPP or amylin) is found to be heavily glycated and to form toxic amyloid-like aggregates, similar to those observed for the Aβ peptides, often also heavily glycated, observed in Alzheimer patients. Here, we studied the effects of glycation on the structure and aggregation properties of IAPP with several biophysical techniques ranging from fluorescence to circular dichroism, mass spectrometry and atomic force microscopy. We demonstrate that glycation occurs exclusively on the N-terminal lysine leaving the only arginine (Arg11) unmodified. At variance with recent studies, we show that the dynamical interplay between glycation and aggregation affects the structure of the peptide, slows down the aggregation process and influences the aggregate morphology.

## Introduction

Glycation is the result of the non-enzymatic covalent attachment of a sugar to a protein, as opposed to protein glycosylation that is an enzyme-mediated reaction. Glycation can occur at the N-terminal group of a poly-peptide or at the side chains of lysines, arginines, cysteines, and histidines (Hipkiss et al., 1995). This post-translational modification is well-known to play a significant role in modifying protein structure and functions, as reported for a broad range of folded proteins (Sambataro and Pennuto, 2017). It is also expected to have a drastic effect on the biological function and biophysical properties of intrinsically unfolded polypeptides (Zhang et al., 2011; Ma et al., 2019), although much less is currently known on this topic. The reaction proceeds through the Maillard reaction of a protein amino group with a sugar to yield a Schiff base (SB), which undergoes a rearrangement to a so called Amadori (AMR) compound (Ulrich and Cerami, 2001). Subsequently, the compound is subject to decomposition, fragmentation, and condensation which finally lead to production of Advanced Glycation End-products (AGEs). High concentrations of AGEs may induce toxicity via aberrant cross-linking with proteins, binding to cell-surface receptor RAGE (receptor for AGEs) and production of oxygen free radicals (Grillo and Colombatto, 2008; Moldogazieva et al., 2019).

Numerous proteins involved in neurodegenerative diseases such as Alzheimer Aβ, tau, alpha-synuclein, and prions are glycated, suggesting that AGEs encourage the development of disease (Castellani et al., 2001; Li et al., 2012). AGEs are for instance believed to be the causative role in the vascular complications of type-2 diabetes (T2D), a pathology heavily associated to hyperglycemia (high blood glucose). A major accepted cause of T2D is the aggregation of IAPP, a small peptide hormone also known as amylin that is co-produced and co-secreted alongside with insulin from the endocrine beta-cells of the pancreas (Lukinius et al., 1989; Zhu et al., 2016). IAPP is synthesized as an 89-residue pre-prohormone proIAPP is a dynamic molecule with four α-helices that is stored in secretory granules at pH 5.5 (Knight et al., 2008; DeLisle et al., 2020), an environment that encourages the protonated state of His18 and contributes to stabilization of the soluble monomer, due to electrostatic repulsion (Jha et al., 2014). After cleavage of the signal sequence, the 67-residue proform is processed in the Golgi and in the insulin β-cell secretory granules to yield the mature hormone. The mature 37-residue peptide requires further processing and post-translational modifications such as amidation of the C-terminus and formation of an intramolecular disulfide bridge between Cys2 and Cys7. Mature IAPP is widely regarded as a natively unstructured protein (Jaikaran and Clark, 2001; Padrick and Miranker, 2001) highly prone to aggregation and formation of insoluble cytotoxic amyloid aggregates and fibrils (Lorenzo et al., 1994; Beck Erlach et al., 2019; Chaari and Ladjimi, 2019; Godin et al., 2019; Palato et al., 2019). Independent evidence has pointed to the region between His18 and Asn21 as critical in the *in vitro* self-assembly of the peptide (Godin et al., 2019; Akter et al., 2020; Ridgway et al., 2020).

*In vitro*, IAPP aggregates rapidly at micro molar concentrations whereas *in vivo* the peptide fails to aggregate at millimolar concentrations (Khatun et al., 2019). Thus, interactions with other constituents of the densely packed granules (C-terminal insulin, zinc ions, IAPP itself) and/or the low pH of granules have been suggested to be physiological inhibitors of IAPP aggregation (Rodriguez Camargo et al., 2017). There is evidence that presence of zinc coordinating with IAPP in the insulin granules plays a critical role in the packaging of insulin but the inhibition of IAPP aggregation as well (DeToma et al., 2012; Tomasello et al., 2015; Ilitchev et al., 2018). Interestingly, peptides derived from IAPP cross-amyloid interaction surface with Aβ are potent inhibitors of Aβ amyloid self-assembly. One of these peptides, R3-GI, was recently found to adopt a β-like structure and oligomerize into colloid-like assemblies in a process that is reminiscent of liquid–liquid phase separation (Niu et al., 2020).

IAPP is the primary constituent of amyloid deposits in the pancreatic islets. In patients affected by T2D, IAPP undergoes conformational changes to form highly-ordered β-sheets organized into amyloid fibers similar to those found in Alzheimer disease (AD) (Sun et al., 2019). The extent of beta-cell loss is heavily correlated with IAPP aggregation: over 90% of T2D patients have extracellular accumulation of IAPP aggregates (Cooper et al., 1987; Kahn et al., 1999, 2014). *In situ* investigations demonstrated that IAPP aggregates extracted from human pancreatic are heavily glycated (Ma et al., 2000). This evidence strongly suggests the potential importance of glycation on IAPP behavior and the need of further studies to clarify its role.

Here, we present a study aimed at understanding the effects of glycation on the structure and aggregation properties of IAPP as probed by circular dichroism (CD) and fluorescence spectroscopies and atomic force microscopy (AFM). We selected methylglyoxal (MGO) as the glycation agent, a short, open-chained dicarbonyl compound. MGO, a metabolite of glucose, primarily reacts with arginine or lysine residues and is estimated to be 20,000–50,000 times more reactive than glucose (Rabbani and Thornalley, 2008; Schalkwijk, 2015; Annibal et al., 2016). MGO-modified peptides can rapidly generate AGEs. Our work parallels and directly complements a recent study in which the authors did not question whether and where MGO glycation of IAPP occurs and chemically synthesized glycated IAPP by replacement of Lys1, the only Lys residue, with *N*ε-(carboxymethyl)-L-lysine to mimic the consequence of protein glycation reaction (Hsu et al., 2019). The peptide formed amyloid faster than unmodified IAPP and higher-molecular-weight AGE-IAPP oligomers were also observed in the early stage of aggregation. These studies demonstrated that glycation modification of IAPP promotes the amyloidogenic properties of IAPP and may play a role in accumulating additional amyloid during T2D progression but offered a static and fait-accompli picture of the effects of IAPP glycation. We reasoned instead that it is important to consider how a post-translational modification modifies the behavior of a peptide while the reaction is still taking place to understand the interplay between glycation and aggregation. We demonstrate that, at variance with this previous study, glycation affects the aggregation process by slowing down the aggregation process and influencing the pathway of aggregation of the peptide. Our results offer an important framework to understand how post-translational modifications change drastically the intrinsic properties of proteins and peptides.

## Materials and Methods

### MGO Preparation

MGO was produced by hydrolysis of pyruvaldehyde dimethyl acetal, according to previously reported procedures (Kellum et al., 1978). Briefly, 10 μl of pyruvaldehyde dimethyl acetal (8,0M) were incubated with 10 μl of sulfuric acid and 80 μl of water at 100°C for 15 min. The reaction progress was monitored by HPLC. 5 μl of the reaction mixture were diluted with 20 μl of pure water and loaded on a Kinetex C18 column (2.1 × 100 mm, 5 μm; Phenomenex). Different compound elution was obtained using a linear gradient from 2 to 20% CH_3_CN in 10 min.

After reaction, the identity of MGO was confirmed by NMR spectra recorded at 25°C on a Bruker AVANCE operating at 800 MHz. The NMR resonances were tentatively assigned by comparison with their predicted chemical shifts (Scifinder CAS registry nr 67-56-1). Pyruvaldehyde dimethyl acetal contains three hydrogens belonging to the methyl group in alpha to the carbonyl group at 2.18 ppm, a single hydrogen in alpha to the two oxygens at 4.44 ppm, and two groups of hydrogens at 3.40 ppm. A potential impurity which did not change in time was detected at 1.45 ppm. At the end of the hydrolysis, the reaction produced two molecules of methanol per one of MGO. The spectrum contained three resonances at 2.38, 4.82, and 5.40 ppm. The presence of two signals for hydrogen B is due to a mixture of two MGO forms. MGO undergoes a spontaneous reaction with water and forms mono- and bi-hydrated MGO (Nemet et al., 2004; Donarski et al., 2010; Behbahani, 2014). Since >99% MGO reacts with water, the only hydrogen belonging to the unreacted aldehyde could not be detected (Lo et al., 1994). The signal of methanol was assigned to the resonance at 3.4 ppm. We could conclude that the reaction was successfully completed from the three signals attributed to MGO at 2.38, 4.82, and 5.40 ppm. The satisfying outcome was supported by the absence of pyruvaldehyde dimethyl acetal associated signals.

### Sample Preparation

IAPP peptides were purchased from Peptide 2.0, Inc. and Eurogentec at >98 and >95% purity respectively as judged by HPLC (using a C18 column, and detection at 220 nm). The peptides arrived as lyophilized powders and were stored at −20°C until use. The samples contained the physiological modifications: a disulfide bridge between Cys2 and Cys7 and the C-terminal amidation as validated by mass spectrometry and reverse-phase HPLC.

The different aliquots of the peptides (1 mg) were always pre-treated under strong acidic conditions with pure trifluoracetic acid (TFA), 1,1,1,3,3,3-Hexafluoro-2-propanol (HFIP) or a combination of both to dissolve pre-existent fibrillar aggregates (Crescenzi et al., 2002). The solvent was added to the sample, mixed vigorously and visually inspected for efficient solubilization. After 1 h incubation at room temperature, the solvent was removed by overnight freeze-drying. The pre-treated protein was then dissolved in phosphate buffer solution 20 mM phosphate at pH 7.4 and 150 mM NaCl to the desired concentration. MGO was added in molar excess and the mixture incubated at 37°C.

### Mass Spectrometry

Glycated IAPP was obtained by adding 5 μl of the described mixture of freshly prepared MGO to 145 μl of PBS containing 80 nmol of IAPP (IAPP:MGO theoretical molar ratio 1:5, 1:10, 1:20, and 1:100) and the reaction was let to proceed for 48 h at 37°C. Reaction products were then analyzed by nanoLC-hrMS/MS using a LTQ Orbitrap XL ESI-mass spectrometer (Thermo Fisher Scientific) equipped with a nano-ESI source, coupled with a nano-Aquity capillary UPLC (Waters): peptides separation was performed on a capillary BEH C18 column (0.075 mm × 100 mm, 1.7 μm, Waters) using aqueous 0.1% formic acid (A) and CH_3_CN containing 0.1% formic acid as mobile phases. Peptides were eluted by means of a linear gradient from 10 to 40% formic acid in 45 min and a 300 nl/min flow rate. Mass spectra were acquired over an *m*/*z* range from 400 to 1800. Mass spectra calibration was performed using NaI clusters as external standard and human [Glu]-Fibrinopeptide B (Sigma-Aldrich, Milan, Italy) as lock mass standard. MGO modified IAPP species underwent MS/MS analysis to confirm the residues involved in the glycation reaction.

### Fluorescence Spectroscopy

Concomitant aggregation and glycation kinetics were followed by spectrofluorometric assays, using a FLUOstar OMEGA Lite instrument. High throughput aggregation kinetics were performed in a Greiner UV-clear 96-well plate either using 10–60 μM peptide samples in 20 mM phosphate buffer at pH 7.4 with and without 150 mM NaCl in the presence and absence of MGO, to obtain peptide:MGO molar ratios 1:0, 1:5, 1:10: 1:20, and 1:100. In early assays we also added 2% of DMSO, which was omitted in the final experiments. Thioflavin T (ThT) was added to reach a concentration of 20 μM. The temperature was set at 37°C. The readings were performed every 15 min, setting the excitation wavelength at 440 nm and the emission wavelength at 485 nm. The plate was left quiescent between measurements and shaken for 1 s before each reading. No shaking was applied during measurement. Glycation kinetics was followed using the same conditions as for the aggregation assays but setting the excitation wavelength at 340 nm and the emission wavelength at 430 nm. Each experiment was repeated at least five times, each time with triplicates, using different batches of the peptides. The results were normalized according to the corresponding blank and expressed in percentage.

### CD Spectroscopy

Far-UV CD spectra were recorded on a Jasco J-1100 spectropolarimeter (Jasco, Essex, United Kingdom), equipped with a temperature control system, using a 1 mm quartz cell in the far-UV range 195–260 nm, using peptide at 10–30 μM concentrations in 20 mM phosphate buffer, pH 7.4 and 15 mM NaCl. For the measurements in the presence of the glycating agent, a 20-fold excess of MGO was added. Constant N_2_ flush at 4.0 L/min was applied. Raw spectra were corrected for buffer contribution and expressed as mean residue molar ellipticity θ (deg×cm^2^×dmol^–1^). The scanning speed was set to 200 nm/min, digital integration time to 1 s, and the temperature set to 37°C for all experiments. Each spectrum was obtained as an average of 10 scans. No shaking was applied on the samples in between measurements. To ensure reproducibility, all experiments were repeated at least three times on at least two different batches of peptides.

### Measurements of the Fiber Morphology by AFM

AFM was carried out on pre-treated samples dissolved in an appropriate volume of PBS to obtain concentrations of 10, 50, 100, and 200 μM. Samples were incubated at 37°C without shaking and images were acquired at different time points, in the absence and in the presence of excess MGO. Height peak force error images were obtained on a Bruker Multimode 8 microscope with a Nano scope V controller (Bruker UK, Ltd., Santa Barbara, CA, United States). Image data were acquired operating in peak force tapping mode using ScanAsyst Air cantilevers (115 μm nominal length, 25 μm nominal width, nominal spring constants of 0.4 Newtons/m, and typical resonant frequencies of 70 kHz). The ScanAsyst probes have a 2 nm nominal tip radius of curvature. Image data were obtained at peak force frequency of 4 kHz and a line rate of 3 Hz at a resolution of 512 pixels/line. After dilution, 10 μl of sample was loaded onto freshly cleaved mica and incubated for 10 min at room temperature. The excess of liquid was dried off from the mica and rinsed with a gentle flux of filtered milliQ-H_2_O.

## Results

### The Importance of the MGO Source

We initially used commercial MGO, purchased from Sigma (cat. no. M0252). In the literature, MGO is extensively used in enzymatic studies. In most cases, it is obtained by steam distillation of a commercial 40% solution in water (Uotila and Koivusalo, 1975). The low effective concentrations of MGO in water and the addition of acid compounds should prevent MGO polymerization (Kellum et al., 1978). However, it is well-known that MGO can undergo oxidation and side reactions when used in the high concentrations and neutral pHs needed for the glycation reaction (Nemet et al., 2004). This means that the effective MGO content of solutions stored even for a short time at the suggested storage temperature (4°C) is questionable (Kellum et al., 1978).

**FIGURE 1:**
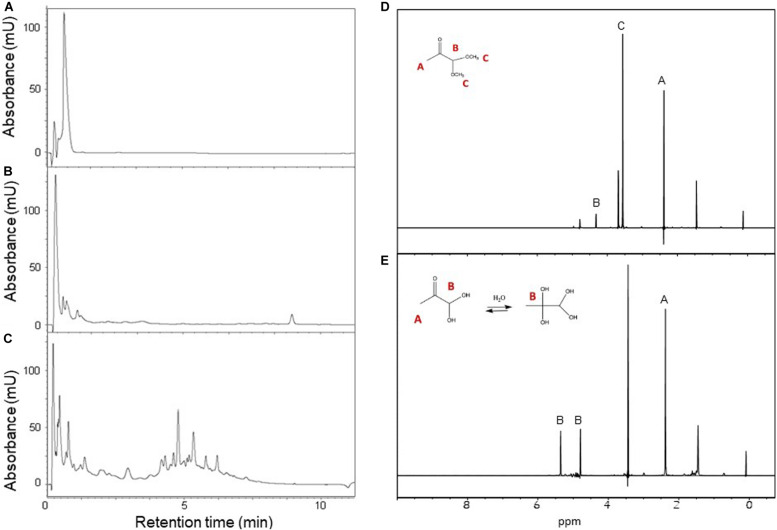
Preparation of MGO starting from pyruvaldehyde dimethyl acetal. Left panel: HPLC-UV analysis of pyruvaldehyde dimethyl acetal hydrolysis products. The reaction mixture was analyzed at the beginning of the reaction **(A)** and after 15 **(B)** and 60 min **(C)** of incubation. Right panel: ^1^H NMR spectra of pyruvaldehyde dimethyl acetal and its product MGO with assignment and their chemical structure. **(D)** Time zero of the reaction, δ: 2.18 (s, 3H, CH3), 3.40 (s, 3H, CH3), 4.44 (s, 1H CH). **(E)** Reaction completed, δ: 2.38 (s, 3H, CH3), 5.40 (s, 1H, CH), 4.82 (s, 1H, CH). The spectra were recorded at 25°C on a Bruker AVANCE spectrometer operating at 800 MHz.

We adopted an alternative, simple, convenient, and reproducible method: high purity MGO was produced from pyruvaldehyde dimethyl acetal under acidic conditions at high temperature in phosphate buffer (Kellum et al., 1978). We used two different acids (HCl and H_2_SO_4_) at different concentrations (from 5 to 25%) and the reactions were monitored over 2 h. The best result was obtained incubating 0.8M pyruvaldehyde dimethyl acetal in 10% H_2_SO_4_ at 100°C for 15 min. Longer incubation times or harsher reaction conditions generated more complex product mixtures, possibly as the consequence of the formation of oligomeric and polymeric species, as previously described. Using this procedure pyruvaldehyde dimethyl acetal was converted in MGO with a nearly 100% yield as established by HPLC and ^1^H NMR (Figure 1).

### Essential Precautions to Obtain Monomeric IAPP

Being aware of our previous experience with Aβ peptides (Emendato et al., 2018), we purchased synthetic peptides spanning the human IAPP sequence from two independent companies (Peptide2.0 and Eurogentec) and compared their behavior to understand the effect of the peptide history on the structure. To dissolve possible pre-existing aggregates and start from a purely monomeric form, we pre-treated different aliquotes of the two peptides with either HFIP, a strong acid alcohol often used in the literature on aggregation-prone peptides (Higham et al., 2000; Abedini et al., 2016; Paul et al., 2017), or with TFA, which offers an even stronger acidic treatment. IAPP in HFIP or TFA (1 ml/mg peptide) was left for 1 h at room temperature before solvent removal by freeze-drying overnight. The peptides, in the form of thin films, were dissolved in buffer and analyzed by far-UV CD to compare their secondary structure.

**FIGURE 2:**
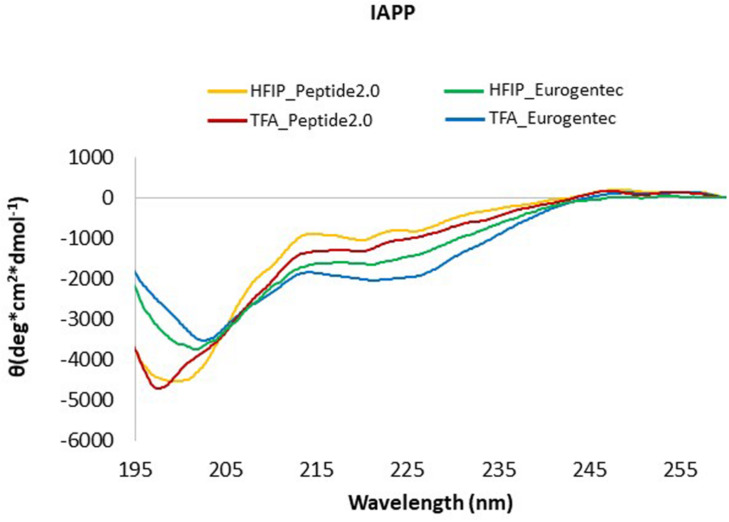
CD Spectra of IAPP from different commercial sources. IAPP from Peptide2.0 pre-treated with HFIP (yellow line) or with TFA (red line); IAPP from Eurogentec after treatment with HFIP (green line) or with TFA (blue line). The spectra were acquired at 25°C in phosphate buffer pH 7.4.

While the choice of solvent did not make much difference, the spectra of the peptides from different source were slightly different (Figure 2). The difference is likely to derive from different purification processes, and, consequently different side products and impurities. IAPP from Peptide2.0 displayed an acceptable CD profile with a higher content of random coil, irrespective of the solvent used for the pre-treatment. However, the peptide pre-treated with TFA showed a minimum at 198 nm (Greenfield, 2006), while, when pre-treated with HFIP, we registered a shift to 202 nm. In all cases, we observed a second weak minimum around 222 nm which might be indicative of some residual helical structure which seemed to be more populated for the peptide from Eurogentec. For these reasons, we decided to use the IAPP synthesized by Peptide2.0 for further studies and we consistently pre-treated it by TFA. We got better yields by first incubating the peptide with pure TFA and then diluting the solution with water (1:10). This treatment reduced adsorption of the peptide to the cuvette walls and allowed us to retrieve it as a powder rather than a film.

We also found that a robust treatment and full elimination of the solubilizing agent is an absolute requirement to study aggregating peptides starting from their monomeric, unfolded form. These results confirm the importance of using synthetic peptides from different sources when it is not possible to have full control on their production.

### Lys1 but Not Arg11 or His18 Is Involved in the Glycation Reaction

Amongst the possible products of MGO-induced glycation is the formation of argpyrimidine, caused by reaction to arginine residues and the lysine-derived AGE produces *N*ε-carboxymethyl-lysine (CML) and *N*ε(1-carboxyethyl) lysine (CEL) (Rabbani and Thornalley, 2008; Gkogkolou and Bohm, 2012; Freund et al., 2018). Argpyrimidine is the only of the AGE products with fluorescence at 370–550 nm wavelength upon excitation at 340 nm, following a procedure previously described (Praveen et al., 2011; Beisswenger et al., 2012). Glycation could thus in principle be followed by fluorescence: we would expect in this case an intense fluorescence signal with a maximum centered at 400 nm as we had observed in our studies on the glycation of Aβ peptide (Emendato et al., 2018). Rather surprising, we did not observe any fluorescence when attempting to follow the reaction with IAPP (data not shown). These results suggested that Arg11, the only arginine of the IAPP sequence, is not involved in glycation.

**FIGURE 3:**
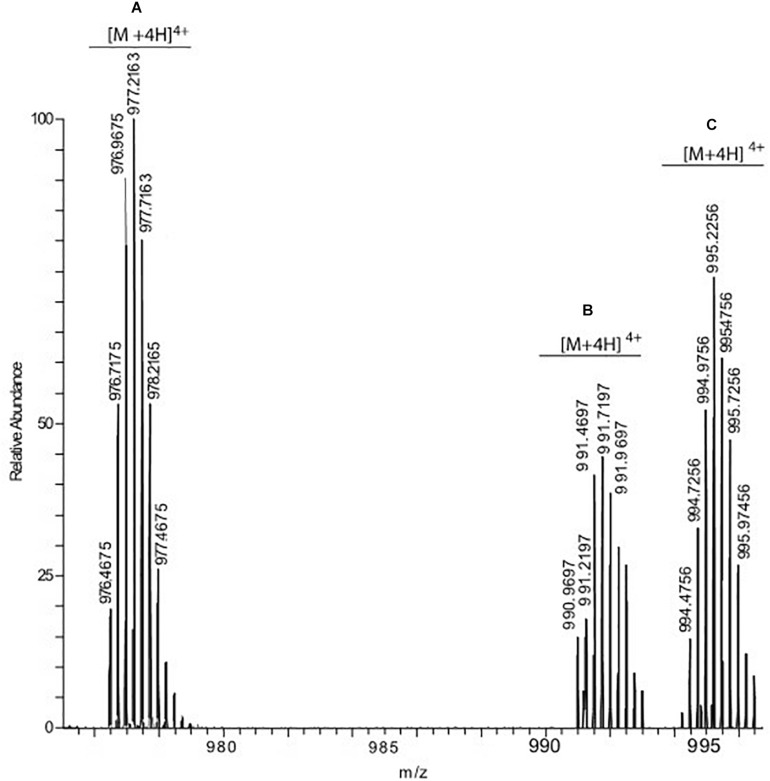
hrESI MS spectrum of IAPP species resulting from the incubation of the peptide with MGO. Three compounds were detected: unmodified IAPP (species A) whose [M + 4H]^4+^ ion was detected at *m/z* 976.4675, IAPP carrying one CML group (species B, [M + 4H]^4+^ at 990.9697) and IAPP carrying one CEL group (specie C, [M + 4H]^4+^ at 994.4756).

To confirm this hypothesis, we used mass spectrometry analysis which can reliably map the modification sites (Emendato et al., 2018). The adducts obtained with arginines or lysines are different and can be distinguished on the basis of the observed mass increment. Modification involving lysines mainly generates CEL (mass increment 72 Da) and CML (mass increment 58 Da), whereas if the reaction involves an arginine residue the reaction products are hydroimidazolone (mass increment 54 Da) and argpyriminide (mass increment 80 Da) (Frye et al., 1998). In our experiments, prolonged (48 h) incubation of IAPP with MGO under mild conditions (MGO:IAPP 10:1 mol/mol; 37°C) induced the formation of two modified species. Mass spectrometry analysis of these species revealed molecular weights of 3973.898 (species C in Figure 3) and 3959.875 (species B in Figure 3), respectively corresponding to a mass increment of 72.032 Da and 58.009 Da as compared to the unmodified peptide (3901.866, specie A in Figure 3). This indicated that the glycation reaction occurs and involves an amine group. In the whole structure of IAPP there are only two reactive amine groups: the N-terminal of the protein and the ε-amine group of Lys1.

The experiment was carried out using different peptide:MGO molar ratios, ranging from 1:1 to a 100-fold molar excess. In all experimental conditions, the only modification site detected was Lys1. The maximal modification yield (about 70%) was obtained using a 10–20-fold molar excess of MGO with respect to IAPP. Higher MGO excesses produced a progressive decrease in the reaction yield that was less than 30% when a MGO:IAPP molar ratio of 100:1 was used. We found anyway completely inappropriate to work at high MGO:peptide molar ratios because MGO tends to acidify the solution and change the pH if in excess and not sufficiently buffered.

These results thus consistently confirm that Arg11 remains unreacted while glycation occurs exclusively at Lys1. Since the two amino groups of Lys1 are located within the same amino acid, it was however impossible to define by mass spectrometry which of the two is the modification site.

### Studies of the Aggregation Kinetics in the Presence and Absence of MGO

ThT-binding assays were employed to follow the aggregation kinetics of IAPP, in the absence or in the presence of increasing concentrations of MGO. We first searched to find the best conditions to have easy-to-follow aggregation kinetics. Different concentrations of peptide and MGO:peptide ratios were screened, ranging from 5 to 60 μM and 0:1 to 100:1 respectively. The kinetics were run in 20 mM phosphate buffer at pH 7.4 that are conditions optimal for the glycation reaction (Emendato et al., 2018). Optimal conditions were found at 10–20 μM IAPP and 10:1–20:1 MGO:peptide ratios. At this peptide concentrations, the aggregation curves were reproducible and the fluorescence signal high enough to define a smooth curve but not as intense to saturate the signal. The aggregation rates were also sufficiently slow to allow observation of a well-defined lag phase.

During an initial screening, we added a small percentage of DMSO (2%) to improve reproducibility. It was previously shown that DMSO does not have appreciable effects on the slope or the lag time of the aggregation of Aβ42 (Habchi et al., 2016). We also noticed that our results were independent from the presence of DMSO (data not shown), but in the kinetics presented here we preferred not to use this co-solvent since its presence constitutes anyway a solvent perturbation.

**FIGURE 4:**
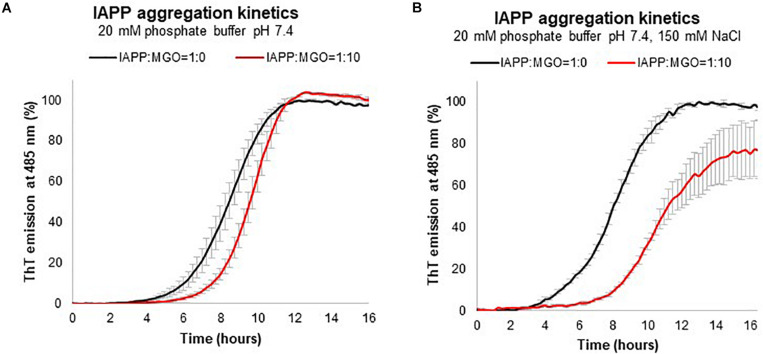
Kinetics of aggregation measured by the variation of the fluorescence signal of ThT. The experiment was carried out using 20 μM IAPP in 20 mM phosphate at pH 7.4 with (red) and without (black) MGO recorded in the absence **(A)** and presence **(B)** of 150 mM NaCl.

The kinetics of aggregation of the IAPP in the absence of MGO was described by a sigmoid, with a lag phase of ca. 4 h in the absence of salt (Figure 4A). The curve reached a plateau after approximately 8 h. Addition of MGO (10:1 MGO:peptide ratio), lengthened the lag phase to 5 h with the plateau reached after 10 h. No marked effect was observed on the elongation phase or with the rate of aggregation as the curve runs parallel to that without MGO. When we repeated the kinetics in the presence of NaCl (150 mM), the lag phase increased to 8 h and the plateau was reached after ca. 16 h (Figure 4B). At these salt concentrations, we also observed an effect on the elongation phase and on the plateau level which is lower in the presence of MGO as it could be expected from the interference between aggregation and the glycation reaction. Increasing peptide concentrations leads to much steeper sigmoids (data not shown), while increasing the MGO:peptide ratios decreased the elongation phase but also the efficiency of glycation so that the plateau decreases so that, at a very high MGO excess (100:1), the plateau is one fifth that observed without MGO (Supplementary Figure S1). At these values, however, MGO is in such an excess that it acts as a co-solvent.

Thus, these results indicate that glycation has an inhibitory effect on IAPP aggregation. MGO affects the kinetics of formation of a critical nucleus and thus acts on the lag phase. These conclusions are independent from the batch of peptide used, the presence or absence of DMSO, and the peptide concentration within the range of concentrations used in the present study.

### Effect of Glycation on IAPP Secondary Structure

The effect of glycation on the secondary structure of the peptide was then monitored by CD spectroscopy. IAPP (20–60 μM) in phosphate buffer (20 mM sodium phosphate, 15 mM NaCl) at pH 7.4 and 37°C with or without a 10 or 20-fold molar excess of MGO. We kept some salt in the buffer but reduced its concentration to minimize interferences with the CD spectrum at low wavelengths.

**FIGURE 5:**
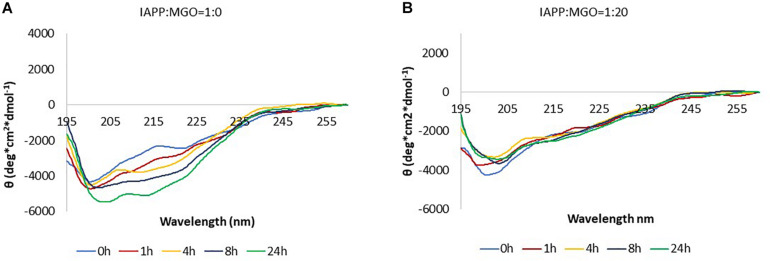
Kinetics of aggregation of IAPP followed by CD. The data were collected in the absence **(A)** and in the presence **(B)** of MGO. The data were acquired at 37°C, on 20 μM peptide and with a 20-fold molar excess of MGO. No shaking was applied in between measurements.

In the absence of MGO, the peptide (20 μM) presented a strong absorption band at ca. 200 nm immediately after dissolving IAPP as described above. This band is characteristic of a high content of random coil (Figure 5A). In the following 4 h. a new minimum at around 215 nm starts appearing. In the following 4 h. a new minimum at around 215 nm starts appearing. After 8 h, the minimum moved toward higher wavelength, suggesting an increment in β secondary structure as also qualitatively supported by spectral deconvolution (Table 1).

**TABLE 1:** Secondary structure content estimated from deconvolution of the CD data.

	IAPP				IAPP + MGO			
	α-Helix %	β-Strand %	Turns %	Random coil %	α-Helix %	β-Strand %	Turns %	Random coil %
0 h	5.3 ± 2.1	36.0 ± 3.6	17.7 ± 5.7	41.3 ± 9.0	8.3 ± 3.2	18.8 ± 4.6	18 ± 7.8	46.3 ± 15.0
1 h	3.6 ± 2.9	42.0 ± 2.1	17.3 ± 6.0	37.0 ± 5.2	6.7 ± 2.1	30.6 ± 5.1	18.3 ± 5.9	44.3 ± 12.1
4 h	2.3 ± 3.2	43.0 ± 3.1	17.3 ± 5.5	38.0 ± 4.6	6.7 ± 2.1	30.6 ± 5.2	18.3 ± 5.1	44.3 ± 12.1
8 h	3.0 ± 2.0	41.0 ± 2.5	17.7 ± 5.8	38.3 ± 4.2	6.7 ± 2.1	30.6 ± 5.3	18.3 ± 5.1	44.3 ± 12.1
24 h	4.3 ± 2.1	42.0 ± 2.0	17.0 ± 5.3	36.7 ± 3.5	7.3 ± 2.3	30.6 ± 5.4	17.7 ± 6.4	44.3 ± 12.1

In the presence of MGO, the peptide displayed a completely different behavior under otherwise the same conditions. Immediately after dissolving the peptide in buffer and adding the glycating agent, the spectrum was consistent with that of non-glycated IAPP at time 0. With prolonged incubation at 37°C, glycation seemed to hinder formation of β-rich structures (Figure 5B). The overall signal intensity remained the same throughout the experiment.

When the experiment was repeated at higher peptide concentration (60 μM), the spectra of the non-glycated peptide followed the same trend as at lower concentration, whereas the glycated peptide eventually aggregated and precipitated subtracting intensity to the signal but without transition toward a β-enriched conformation (Supplementary Figure S2).

These data indicate that the presence of MGO, together with the concomitant glycation of the peptide, slows down the aggregation pathway of IAPP.

### Analysis of the Aggregate Morphology

We then used high-resolution AFM to obtain information on the morphology of the species formed at different time points in the presence and absence of MGO. Samples of IAPP and glycated IAPP (initial concentration of 10, 50, 100, and 200 μM) were analyzed after 3 day incubation at 37°C.

**FIGURE 6:**
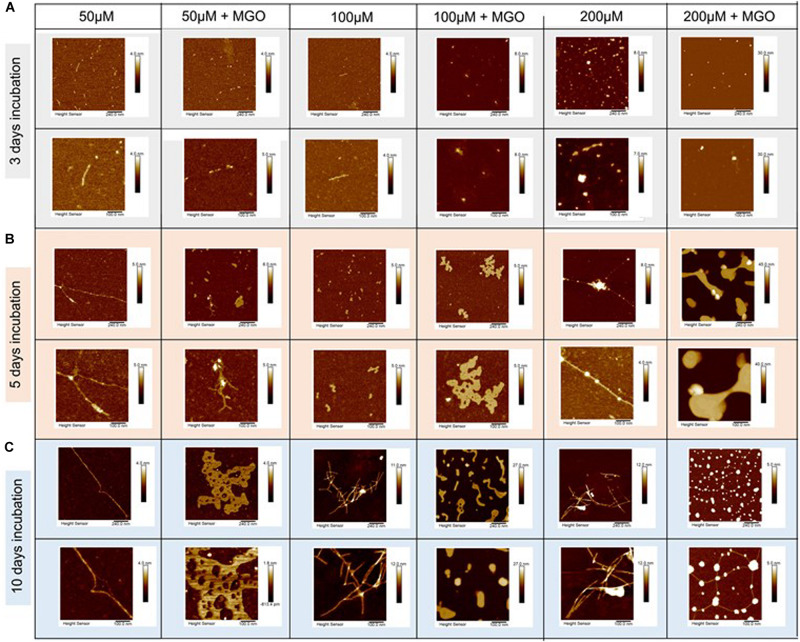
Effects of glycation of IAPP on the aggregate morphology studied by atomic force microscopy. AFM micrographs of IAPP at concentrations of 200, 100, and 50 μM incubated with 20-fold ratio MGO in PBS at 3 day **(A)**, 5 day **(B),** and 10 day **(C)** incubation. IAPP images are displayed from a 5 μm scale (top row) to 500 nm scale (bottom row). The images show that glycation interferes.

The micrographs collected at 10 μM showed only few oligomers/protofibers also after 5 days indicating slow kinetics (Supplementary Figure S3). We thus decided to work with more concentrated samples in which aggregation would be enhanced. The micrographs of IAPP without MGO at 50 μM peptide contained discoidal oligomers with the occasional appearance of some pre-fibrillar specie (Figure 6 and Table 2). Their height increased from less than 1 nm after 3 days of incubation to 1.3 nm after a 5 day incubation. These fibrillar species became progressively longer at longer pre-incubation times becoming needle-like at 10 days. Their shape was slightly twisted, characterized by two heights along the fibers: 1.5 and 1.2 nm. The corresponding glycated samples contained similar but sparser oligomers at 3 days. These evolved to form branched rare needle-like species with a 1.3 nm high at 5 days, characterized by spreaded aggregates almost 7.5 nm high. At 10 days, wreath-shaped bundles of bulky aggregates became visible.

**TABLE 2:** Summary of the heights of the glycated and non-glycated IAPP fibers as obtained by AFM.

IAPP concentration	3-day incubation		5-day incubationh		10-day incubation	
	IAPP	IAPP + MGO	IAPP	IAPP + MGO	IAPP	IAPP + MGO
50 μM	0.999 ± 0.050 nm		1.338 ± 0.100 nm	1.246 ± 0.050 nm	2 heights along fiber	1.156 ± 0.100 nm
				Blob = 7.409 ± 0.200 nm	1.518 ± 0.050 nm	
					1.189 ± 0.050 nm	
100 μM	1.032 ± 0.010 nm	4.139 ± 0.050 nm	1.324 ± 0.450 nm	1.801 ± 0.250 nm	2 heights on fiber	13.301 ± 0.400 nm
					4.370 ± 0.100 nm	
					8.507 ± 0.100 nm	
200 μM	3.5 ± 0.050 nm	0.980 ± 0.150 nm	Fiber = 1.12 ± 0.050 nm	20.20 ± 0.200 nm	3 heights along – corresponds to different areas of twisting along fiber	1754 ± 0.100 nm
	Blob = 14.89 ± 0.050 nm	Blob = 12.88 ± 0.150 nm	Blob = 4.06 ± 0.150 nm		3.688 ± 0.200 nm	8044 ± 0.300 nm
					5.072 ± 0.250 nm	Highest point of ‘fiber’ = 10.33 ± 0.200 nm
					8.315 ± 0.200 nm	

*Heights are displayed for each time point and each protein concentration. N.d., not determined.*

At peptide concentration of 100 μM, we observed a similar picture: small pre-fibrillar species (1.0 nm high) were observable after 3 days incubation for the non-glycated peptide. The pre-fibers evolved into thin fibers characterized by a height of 1.3 nm after 5 days incubation. These thin fibers progressed into long needle-like branched and dense fibers at 10 days incubation. These fibers displayed a twisted shape with heights of 4.4 nm 8.5 nm corresponding to the two groves of the twist. Conversely, the glycated peptide did not result in fibrillary species at 3 day incubation, but after a 5 day incubation they produced bundling wreath-like aggregates with a height of 1.8 nm. This morphology was similar to the one of the aggregates observed for the 50 μM sample after longer incubation. After 10 days, the aggregates evolved into large species strikingly different from those observed for the non-glycated IAPP. These aggregates were characterized by very thick consistence and a 14.3 nm height.

Finally, when analyzing samples at a concentration of 200 μM, for non-glycated IAPP we observed a clear evolution from discoidal oligomers at 3 days to isolated needle-like fibers at 5 days. These fibers became highly branched after 10 days incubation. The height of the fibers increased from 1.1 nm at 5 day incubation to thicker and more twisted fibers characterized by three different areas of twisting along fiber: 3.7, 5.7, 8.3 nm at 10 day incubation.

The glycated peptide developed from small punctuated oligomers of 0.981 nm height at 3 days to large shapeless aggregates of 20.2 nm height at 5 days, similar in shape and thickness to the glycated aggregates observed at 100 μM after longer incubation. At 10 day incubation, the glycated IAPP at 200 μM evolved into discoidal aggregates of 10.3 nm height linked by 1.7 nm-high fibers. The results were qualitatively reproducible in the presence (Figure 6) and absence of DMSO (data not shown).

This behavior supports the evidence that glycation induces IAPP to adopt a different aggregation pathway, interfering with the kinetics of its fibrillation. The final fibrils formed by glycated and non-glycated IAPP showed a similar morphology, despite the very different pathway.

## Discussion

The IAPP hormone is an important target because of its role in T2D. Here, we studied how glycation interferes with the aggregation properties of IAPP. Using synthetic peptides and a combination of spectroscopic and biophysical techniques, we demonstrated that MGO glycation of IAPP results in the formation at Lys1 of CML or CEL, two of the most common AGE products (Fu et al., 1996; Frye et al., 1998; Xue et al., 2011). In the initial phases of the project we learned two important lessons. First of all, we understood the importance of peptide history. Peptides from different sources may have different degrees of residual aggregation and need to be pre-treated. Many groups have used for pre-treatment HFIP, which, being volatile, can easily be eliminated by freeze-drying. We found that, while this is true, treatment with the stronger TFA is more secure to obtain acceptably comparable starting molecules. We also found that the molar ratio between the glycating agent and the peptide cannot be exceedingly high. We observed by mass spectrometry that to have maximal yield it is not necessary but actually detrimental to reach high molar ratios of MGO to peptide. The optimal ratio in our case is around a 10–20-fold excess. This is likely because, if too concentrated, MGO acts itself as a co-solvent and helps solubility. This results in the deceiving impression of an inhibitory effect of aggregation that it should instead be ascribed to the MGO still soluble in solution and non-ligated. The reaction never reaches 100% because being rather inefficient, the peptide aggregates before it is glycated thus shielding the glycating sites. At the same time, high concentrations of glycating agents such as 100:1 are more than likely non-physiologic also for T2D patients and can thus be neglected. These observations will need to be kept in mind in future glycation studies.

We observed no production of the MGO-derived products argpyrimidine on Arg11 or hydroimidazolone on His18. This is the first direct evidence showing that glycation occurs only at the first IAPP residue, possibly because it is more accessible to MGO modification. The overall effect of the glycation reaction on IAPP aggregation is inhibitory. Glycation affects aggregation by interfering with the elongation phase dramatically altering the aggregation kinetics by decreasing the slope of the kinetics as measured by ThT-binding assays. This effect is associated with an altered pathway of aggregation which slows down a secondary structure transition from random-to-β and leads to a different morphology of the IAPP aggregates. On the other hand, MGO does not prevent aggregation as we can deduce from the reduction of the ThT-associated signal.

We used AFM to investigate the morphology of IAPP in the presence and in the absence of MGO. This technique is often used to characterize molecular aggregates because has the advantage, as compared to electron microscopy, to work directly in solution. Some care should however be paid in interpreting the results since they depend on the scan image size: AFM can only image a maximum scanning area of ca. 150 × 150 μm and a maximum height on the order of 10–20 μm. The scanning speed of an AFM scan is also a limitation since, being slow, it often leads to a thermal drift of the image. This is why we are using the AFM information mostly qualitatively. The scans we collected clearly indicated that glycation led IAPP toward a different aggregation pathway: non-glycated IAPP displayed fiber formation following a canonical pathway which goes from the monomeric form to proto-fibers to fibrillar aggregates. Glycated IAPP showed an evolution from discoidal aggregates to wreath-like highly interconnected species.

These data should be compared with similar investigations from the literature. Several studies have shown that post-translational modifications on specific amino acid side chains significantly affect IAPP aggregation but with inconsistent outcomes (Dunkelberger et al., 2012; Nguyen et al., 2017). The effect of IAPP deamidation, a spontaneous non-enzymatic post-translational modification resulting in the conversion of asparagine into a mixture of aspartic acid and iso-aspartic acid, was investigated by two different groups. Dunkelberger et al. (2012) demonstrated that IAPP deamidation accelerates IAPP self-assembly, by altering the fibers structure (Dunkelberger et al., 2012). The amino acids responsible were identified to be Asn14 and Asn21 but not Asn22, Asn31, and Asn35 (Nguyen et al., 2017).

More directly comparable with our studies are investigations on the effects of glycation. One of the first studies of IAPP glycation was carried out by Kapurniotu (2001), who reported that glycated IAPP is more amyloidogenic. The different outcome between these studies could be explained by different factors. First, the authors used a different glycating agent, D-glucose, which is up to 50,000 times less reactive than MGO (Rabbani and Thornalley, 2008; Schalkwijk, 2015; Annibal et al., 2016). This implies a quite different balance between glycated and non-glycated species. Second, the authors obtained a different AGE: they described the conversion of Arg11 guanidine group in imidazolone and thus follow a quite different process. They were thus following an alternative pathway. Third, the whole logic was different. The glucose-glycated product (AGE-IAPP) was left in incubation for 4 days and only after added to native IAPP. It is not surprising that in this way AGE-IAPP acted as a seed for the aggregation process. Similarly, a recent study demonstrated exacerbation of IAPP aggregation upon glycation using MGO (Hsu et al., 2019). However, the study answers a question completely different from that formulated in our investigations: these authors chemically synthesized MGO-glycated IAPP in which they replaced Lys1 with *N*ε-(carboxymethyl)-L-lysine to mimic the consequence of protein glycation reaction and followed what the behavior of this specie in time. The glycated peptide formed amyloid faster than unmodified IAPP and higher-molecular-weight AGE-IAPP oligomers. There was non-glycating reaction acting in concomitance. We wanted instead to follow how the very process of glycation, which is presumably what happens when IAPP is released and/or sugar levels arise, affects aggregation. We observed a protection effect. This duality of behavior, aggregation enhancement when starting with an already glycated peptide and inhibition when the aggregation reaction co-occurs suggests also a duality of response depending on the environmental conditions.

We might then wonder why glycated IAPP seems to be more toxic (Kakinen et al., 2019; Ma et al., 2019). The answer could be in a study focused on the relationship between IAPP and sugars carried out in 2000 (Macfarlane et al., 2000). This study demonstrated that sugar has a strong effect on IAPP gene transcription and acts as a regulation of the expression and amounts of IAPP. The authors demonstrated that IAPP oligomers are the most toxic species among the IAPP aggregates and induce membrane leakage and disruption (Haataja et al., 2008). These oligomers are able to cross the plasma membrane and act similarly to prions (Mukherjee et al., 2017; Kiriyama and Nochi, 2018). When glycation slows down the aggregation process, the oligomeric species are maintained for longer time in solution before eventually forming amyloid-like aggregates. This means that glycated IAPP can be more toxic as a consequence of a longer persistence as oligomeric species.

We can thus conclude that our studies may contribute in understanding how post-translational modifications increase the functional (normal and pathologic) space of proteins and offer a unique possibility to modulate their cellular role.

## Data Availability Statement

All datasets generated for this study are included in the article/Supplementary Material.

## Author Contributions

GM and EZ did all the CD, ThT, and AFM experiments. MP and RP analyzed the data. FD carried out the MS experiments. PT instructed specific experiments and the production of MGO. AP supervised the project. GM wrote the first draft which was finalized by AP. All authors contributed to the manuscript preparation.

## Conflict of Interest

The authors declare that the research was conducted in the absence of any commercial or financial relationships that could be construed as a potential conflict of interest.
