# Supplementary material for: The Role of Glycation on the Aggregation Properties of IAPP
Source: Front Mol Biosci. 2020 Jun 3;7:104. doi: 10.3389/fmolb.2020.00104 (PMC7284065; doi:10.3389/fmolb.2020.00104)

**Supplementary Materials**

**Figure S1 –** ThT assays at varying IAPP:MGO molar ratios. The experiment used 0, 5, 10, 20 and 100-fold molar excesses of MGO to IAPP, using 10 µM IAPP concentration from a DMSO stock. The data were represented as ThT fluorescence intensity at 485 nm and expressed as percentages.

**Figure S2 –** CD experiments of 60 µM IAPP in the absence (A) and the presence (B) of 20-fold excess MGO. CD profiles were recorded at different time points. At this peptide concentration, the shift of IAPP by itself towards β-sheet rich conformations is very pronounced, while the intensity of the signal for the sample incubated with MGO decreases with time, probably due to precipitation phenomena.


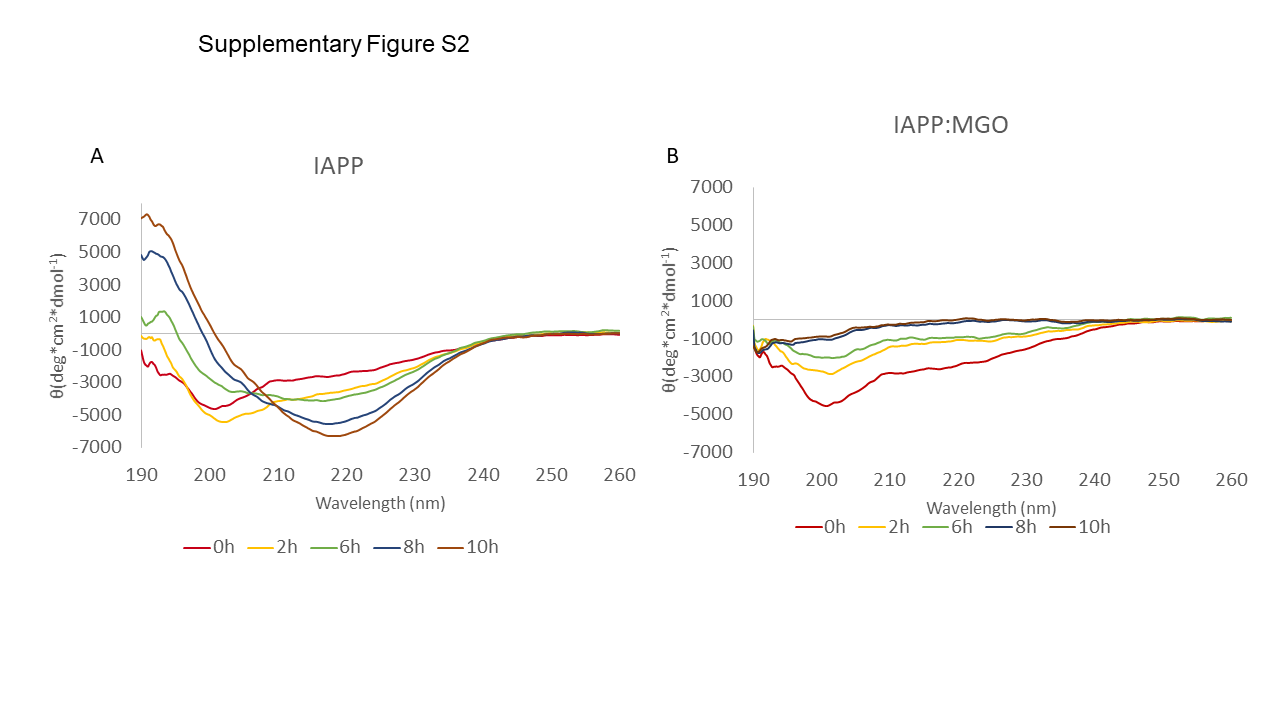


**Figure S3 –** Effects of glycation of IAPP on the aggregate morphology studied by atomic force microscopy. AFM micrographs of IAPP (10 µM) incubated at 37 ºC without shaking for 5 days. IAPP images are displayed from a 5 µm scale (left) to 500 nm scale (right).


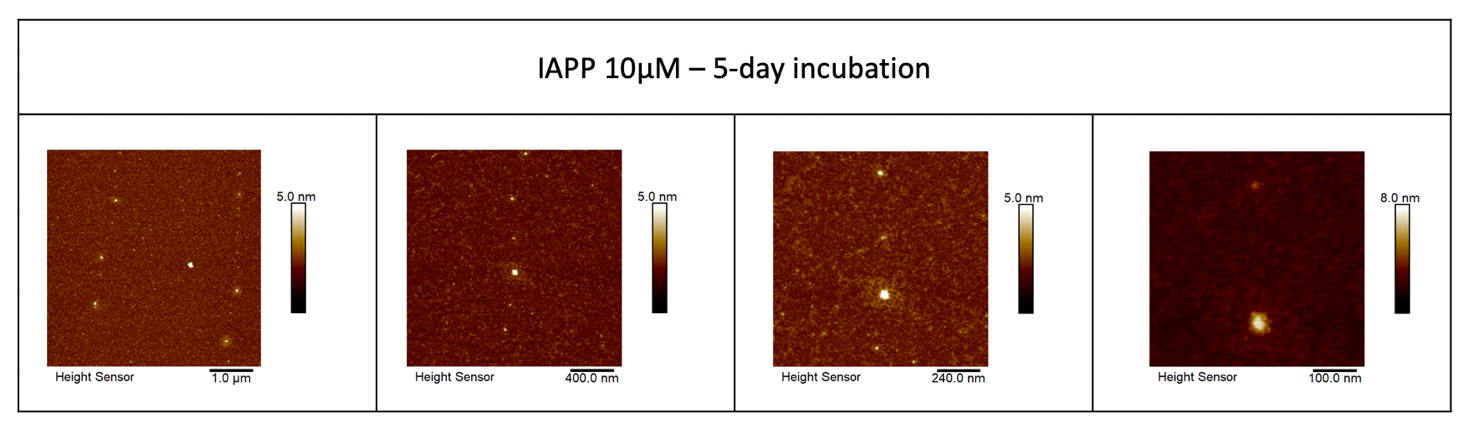

Supplement: Supplementary file 1 [file Data_Sheet_1.docx]
